# Quality management, quality assurance, and quality control in medical physics

**DOI:** 10.1002/acm2.13885

**Published:** 2023-01-19

**Authors:** Max Amurao, Dustin A. Gress, Mary Ann Keenan, Per H. Halvorsen, Jonathon A. Nye, Mahadevappa Mahesh

**Affiliations:** ^1^ Department of Radiation Safety Washington University School of Medicine in St. Louis Saint Louis Missouri USA; ^2^ Department of Quality and Safety American College of Radiology Reston Virginia USA; ^3^ Department of Radiology Vanderbilt University Medical Center Nashville Tennessee USA; ^4^ Department of Radiation Oncology Beth Israel Lahey Health Burlington Massachusetts USA; ^5^ Department of Radiology and Imaging Sciences Emory University Atlanta Georgia; ^6^ Department of Radiology and Cardiology Johns Hopkins University School of Medicine Baltimore Maryland USA

**Keywords:** medical physics, quality assurance, quality control, quality management

## Abstract

The historic and ongoing evolution of the practice, technology, terminology, and implementation of programs related to quality in the medical radiological professions has given rise to the interchangeable use of the terms Quality Management (QM), Quality Assurance (QA), and Quality Control (QC) in the vernacular. This White Paper aims to provide clarification of QM, QA, and QC in medical physics context and guidance on how to use these terms appropriately in American College of Radiology (ACR) Practice Parameters and Technical Standards, generalizable to other guidance initiatives. The clarification of these nuanced terms in the radiology, radiation oncology, and nuclear medicine environments will not only boost the comprehensibility and usability of the Medical Physics Technical Standards and Practice Parameters, but also provide clarity and a foundation for ACR's clinical, physician‐led Practice Parameters, which also use these important terms for monitoring equipment performance for safety and quality. Further, this will support the ongoing development of the professional practice of clinical medical physics by providing a common framework that distinguishes the various types of responsibilities borne by medical physicists and others in the medical radiological environment. Examples are provided of how QM, QA, and QC may be applied in the context of ACR Practice Parameters and Technical Standards.

## 1 INTRODUCTION

A fundamental tenet in the practice of medicine involves maximizing clinical benefit to the patient, while minimizing associated risks to the patient including their caregivers. In the diverse applications spanning radiology, radiation oncology, nuclear medicine and molecular imaging, medical physics, and various imaging‐guided medical practices, implementing this tenet may involve optimizing diagnostic image quality, therapeutic gain, and/or image guidance accuracy (relevant to planning treatments or delivering medical procedures). These goals directly emphasize the critical importance of quality and safety programs, which are cornerstones for the American College of Radiology (ACR). To this aim, the ACR Commission on Quality and Safety provides oversight and management for all radiology quality and safety programs and initiatives, including Practice Parameters and Technical Standards, Appropriateness Criteria®, accreditation programs, centers of excellence, quality measurements, National Radiology Data Registry, RADPEER™ and Imaging‐RADS.^1^

The historic and ongoing evolution of the practice, technology, terminology, and implementation of programs related to Quality in the radiological sciences has given rise to the interchangeable use of the terms Quality Management (QM), Quality Assurance (QA), and Quality Control (QC) in the vernacular. AAPM Report 283 (Task Group 100)^2^ presented a well‐organized and in‐depth discussion on QM, QA, and QC, coupled this discussion with risk analysis methods, and applied it with impressive detail for Intensity Modulated Radiation Therapy (IMRT). The two primary objectives of this White Paper are (a) to re‐present an overview on the use of these three terms, as well as (b) to provide examples of how QM, QA, and QC may be applied in medical physics broadly, and particularly in ACR's Practice Parameters and Technical Standards. This White Paper is a work product of ACR's Committee on Practice Parameters – Medical Physics, which is under the auspices of ACR's Commission on Medical Physics. This White Paper represents a position of the ACR.

Technical Standards describe technical procedures or practices that are quantitative or measurable. These often include recommendations for equipment specifications or settings, and are intended to set a minimum level of acceptable technical proficiencies and equipment performance.^3^ Practice Parameters describe recommended conduct in specific areas of clinical practice.^4^ ACR Technical Standards and Practice Parameters are based on the analysis of current literature, expert opinion, open forum commentary, and formal consensus. It is important to emphasize that the recommended standards and parameters are not intended to be legal standards of care or conduct; and may be modified as determined by individual circumstances and available resources. Notably, there are over 20 Medical Physics Technical Standards and Practice Parameters to date.

Summary of terms and definitions:

QM: An overall management system that includes establishing quality policies and quality objectives, and processes to achieve quality objectives through quality planning, QA, QC, and quality improvement.

QA: A component of QM focused on providing confidence that quality requirements will be fulfilled; it includes all activities (planned, systematic, and practice‐based activities) that demonstrate the level of quality achieved by the ***output*** of a process.

QC: A component of QM focused on the fulfillment of quality requirements; it includes activities that impose specific quality on a process; and entails the evaluation of actual operating performance characteristics of a device or system, comparing it to desired goals, and acting on the difference; QC works on the ***input*** to a process to ensure that important elements or parameters specific to the process are correct.

### 1.1 Quality management

The broad description of QM (according to ISO 9000), is that it can include establishing quality policies quality objectives, and processes to achieve these quality objectives through quality planning, QA, QC, and quality improvement.^5^

A QM program will be comprised of many components that may include: radiation monitoring, management of radioactive sources, incident learning, treatment process QA, equipment and system QA, and equipment QC. Ideally, a QM program must be established for each planned, systematic, practice‐based activity and should include hazard analysis, QC, QA, training and documentation, and ongoing quality improvement efforts.

Reactive QM is commonly performed in response to a QA‐detected failure or an adverse event, often taking the form of a “root‐cause‐analysis” as part of an incident investigation. Prospective QM is ideal, where timely intervention on process inputs (QC) and/or process outputs (QA) are implemented before the pre‐determined criteria for quality is exceeded. Risk analysis methods such as process mapping, Failure Modes and Effects Analysis, and Fault Tree Analysis are well‐developed approaches for prospective QM. A typical data‐driven strategy for Quality Improvement is the DMAIC algorithm (Define, Measure, Analyze, Improve, and Control) often incorporated in a “six‐sigma” initiative.

According to the American Society for Quality, QM involves managing activities and resources of an organization to achieve objectives and prevent nonconformances; while a Quality Management System (QMS) is a formal system that documents the structure, processes, roles, responsibilities and procedures required to achieve effective QM.^37^ Effective QM and QMS requires active collaboration among all members of a multi‐disciplinary team, including physicians, technologists, nurses, dosimetrists, medical physicists, administrators, and service engineers, among others. This is commonly achieved through the structure of a Quality and Safety (Q&S) Committee, such as a Radiation Safety Committee, MRI Q&S Committee, or Patient Q&S Committee.

### 1.2 Quality assurance

The broad description of QA (from ISO 9000) is that it is a component of QM focused on providing confidence that quality requirements will be fulfilled.^5^ QA includes all activities (planned, systematic, practice‐based) that demonstrate the level of quality achieved by the *output* of a process.^6^ As defined by the International Electrotechnical Commission, a process is a set of inter‐related resources and activities that transform inputs into outputs. QA assesses the correctness of the process output, after taking relevant inputs into account, including equipment, systems, and procedures.^2^ Although the various ACR Technical Standards and Practice Parameters are in different stages of explicitly identifying QA, relevant parameters quantifying the output of a process are regularly used in clinical practice. Examples of the relevant parameters related to various ACR Technical Standards and Practice Parameters are included in Table 1.

**TABLE 1:** Examples of QA and performance criteria for select ACR technical standards and practice parameters

**Technical standard or practice parameter**	**Example of quality assurance**	**Performance criteria**
Computed tomography (CT) equipment^7^	Review of clinical protocols to ensure appropriate technique parameters to include kV, mAs, detector configuration, and pitch to ensure protocols meet ACR recommended limits for CTDI_vol_ values and other relevant dose metrics as compared to established reference values.	“The Qualified Medical Physicist must review the most commonly used protocols to include head and abdomen protocols for adult and pediatric patients as well as potential high‐dose procedures (e.g., brain perfusion)^7^.” CTDI_vol_ values should not exceed ACR CTAP Reference Values, and must not exceed the ACR CTAP Pass/Fail Values.^7^, ^8^
External beam therapy^9^	Review of the treatment plan (pre‐treatment), verification at the first treatment session, weekly review of on‐treatment patient charts, and review at end‐of‐treatment to confirm the delivery of the treatment plan within acceptable thresholds.	The pre‐treatment plan review should involve verification of parameters that are used to describe the radiation beam(s) used in the treatment plan. These parameters must reflect the intent of the prescription. The day‐one treatment verification reviews should address any element or record of the prescription that is ambiguous, erroneous, or suspected of being erroneous. Any deviations from the treatment plan discovered at the end‐of‐treatment must be documented and promptly brought to the attention of the attending radiation oncologist.
Fluoroscopic equipment^10^	Evaluation and optimization of fluoroscopic patient doses ensuring representative doses do not deviate significantly from accepted Dose Reference Levels and Achievable Doses	A regular review of patient radiation dose indices and a comparison with guidelines identifying contributing factors that can be optimized for better alignment with recommended dose levels.^10^, ^11^, ^12^, ^13^
High dose rate brachytherapy physics^14^	Dose delivery QA at pre‐treatment, and before each treatment fraction verifying that the treatment plan can be delivered within acceptable margins of error.	Prior to each treatment, perform a dummy wire check to ascertain that all catheters/applicators are unobstructed. For multi‐fraction HDR, validation that parameters used for treatment of the first fraction are appropriately corrected for source activity and remain valid for the remaining treatment fractions.
Mammographic equipment^15^	Evaluation of mammographic image reject/repeat analysis program.	Evaluating percentage of rejected images to ensure a threshold of less than 3%–5% , ensuring standardized reasons for rejection are reasonable, developing action plan to optimize reject rates based on the clinical environment^16^
Stereotactic body radiation therapy^17^	“Patient‐Specific QA (PSQA) verifying that the patient's treatment plan can be accurately delivered.”	PSQA instrumentation must be available and calibrated, pre‐treatment PSQA reviews/second‐checks/dry‐runs performed and documented, treatment incidents are documented with proper follow‐up actions.^17^
Therapeutic procedures using radiopharmaceuticals^18^	Administered activity within action levels, and below reporting thresholds for a medical event.	Unless otherwise directed by the authorized user, a licensee may not use a dosage if the dosage does not fall within the prescribed dosage range or if the dosage differs from the prescribed dosage by more than 20%.^19^ The thresholds for a medical event are described in US Nuclear Regulatory Commission regulations.^20^

As discussed later for QC, a important criteria of QA is the establishment of corrective actions to be taken should a process demonstrate a failure of a quality metric. The QMP should advise on when corrective actions should occur, who should provide corrective actions or services, and how to document these actions based on the individual facility's policies, accreditation, or regulatory requirements.

### 1.3 Quality control

The broad description of QC (from ISO 9000) is a component of QM focused on the fulfillment of quality requirements.^5^ It includes activities that impose specific quality on a process, and entails the evaluation of actual operating performance characteristics of a device or system, comparing it to desired goals, and acting on the difference.^6^ Generally, QC works on the *input* to a process to ensure that important elements or parameters specific to the process are correct.^2^ The use of relevant QC techniques (e.g., checklists, run‐charts, time‐outs, etc.) completed prior to performing the procedure is an established and effective practice for maintaining quality and safety. Overseeing and performing QC is a critical part of a Medical Physicist's role, and guidance on modality‐specific QC is described in the appropriate Medical Physics Technical Standard, Practice Parameter, AAPM Medical Physics Practice Guideline, AAPM Task Group Report, or Accreditation/Technical Reference. Examples of QC and the relevant performance criteria related to various ACR Technical Standards and Practice Parameters are included in Table 2.

**TABLE 2:** Examples of QC and performance criteria for select ACR technical standards and practice parameters

**Technical standard or practice parameter**	**Example of parameter measured for quality control**	**Performance criteria**
Computed tomography (CT) equipment^7^	CT number (Hounsfield Unit) accuracy	Verify that indicated CT numbers are accurate based on known values of representative phantom target inserts with respect to parameters and techniques associated with the phantom used. For instance, CT number values for real water should report as 0 ± 5 HU for all kV settings.^8^
Use of radiopharmaceuticals in diagnostic procedures^21^	“The quantity of radioactivity to be administered must be assayed prior to administration.”^21^	Unless otherwise directed by the authorized user, a dosage may not be administered if it does not fall within the prescribed dosage range or if the dosage differs from the prescribed dosage by more than 20%.^19^ The identity of the patient, the radiopharmaceutical, the route of administration must be verified prior to administration and documented in the patient's record.
External beam therapy^9^	Output verification for each beam (energy and type)	Independent verification of the output of each photon and electron beam must be performed to verify that the treatment unit calibration is consistent with national standards.
Fluoroscopic equipment^10^	Accuracy of entrance skin exposure (R/min) (or Air Kerma Rate [mGy/min])	The displayed air kerma at a reference point (Ka,r) value shall not deviate from the actual value by more than ± 35% above 100 mGy, and air kerma‐area product (KAP) shall not deviate from the actual value by more than ± 35% above 2.5 Gy‐cm^2^.^22^, ^23^
Gamma cameras^24^	Intrinsic flood field uniformity	“For each gamma camera head, the system uniformity with all commonly used collimators should be evaluated. Interdetector variability should also be evaluated. Acceptable performance depends on how the gamma camera is used clinically^24^”. “The IU for 5 million count floods over the UFOV should be less than 5%^25^”.
High dose rate brachytherapy physics^14^	Accuracy of source dwell position and dwell time	“The desired mechanical accuracy and precision of ≤ 1 mm must be performed on the HDR source(s) before the first use of the after loader on a given day. Accuracy and linearity of the source dwell time must also be determined^14^”.
Low dose rate brachytherapy physics^26^	Source strength accuracy	Brachytherapy sources must have measurements of their source strength (specified in terms of air kerma strength) with traceability to national standards.
Magnetic resonance imaging (MRI) equipment^27^	Geometric accuracy	Using the ACR Large MRI Phantom, geometric accuracy should be 148 ± 2 mm for the Sagittal Localizer along the Head/Foot direction, and 190 ± 2 mm for the Axial T1W images along the A/P and R/L directions.^28^
PET‐CT equipment^29^	“Accuracy of CT‐based attenuation and scatter correction, and SUV measurement should be evaluated on at least an annual basis.”^29^	Background mean SUV should be between 0.85 and 1.15.25 mm “hot” maximum SUV should be between 1.8 and 2.8, ratio of the 25 mm/16 mm “hot” maximum SUV should be above 0.7.
Mammographic equipment^30^	Determination of Average Glandular Dose (AGD) and assessment of Accuracy of Displayed AGD.	Measurement of entrance skin exposure for an average glandular breast, as defined by FDA, under specified measurement conditions. Calculation of average glandular dose to ensure dose does not exceed FDA limit of 3 mGy. Indicated AGD metric must be within ± 25% of measured dose.^30^, ^31^
Stereotactic/Tomosynthesis‐guided breast biopsy systems^32^	kVp accuracy and reproducibility^32^, ^33^	To assure that the actual kVp is accurate to within ± 5% of the indicated kVp and that the kVp is reproducible as indicated by a coefficient of variation less than or equal to 0.02.
Stereotactic body radiation therapy^17^	Clearly defined pre‐treatment check should be performed, to include: collision check, correct site, correct body markings for treatment site, correct immobilization equipment, prescribed image guidance technique.^17^	Pre‐treatment verification of no collisions, correct site, correct body markings, correct immobilization equipment, and correct image guidance should be performed.^34^
Therapeutic procedures using radiopharmaceuticals^18^	“Accuracy of the dose calibrator response to traceable standards of radioactivity.”^35^	For radionuclide calibrator field instruments, radionuclide calibrator accuracy is recommended to be within ± 5% for photon emitters > 100 keV, within ± 5% for medium‐ and high‐energy beta emitters, and within ± 10% for low‐energy beta emitters. For secondary standard radionuclide calibrators and reference, radionuclide calibrators should be calibrated to within ± 2% for photon emitters > 100 keV and medium and high‐energy beta emitters, and within ± 5% for photon emitters < 100 keV and low‐energy beta emitters.^36^

Another aspect of QC is the establishment of corrective actions to be taken should the device or system fail to meet the desired performance goals or QC criteria. The Qualified Medical Physicist should advise on when corrective actions should occur, who should provide corrective actions or services, and how to document these actions based on the facility's policies, accreditation, or regulatory requirements. See NCRP Report No.99, QA for Diagnostic Imaging Equipment for guidance on setting up a corrective action plan.

## 2 CONCLUSIONS

The framework of QM, QA, and QC may be implemented in a variety of ways, across a vast spectrum of applications spanning Radiology, Radiation Oncology, Nuclear Medicine and Molecular Imaging, Medical Physics, and various imaging‐guided medical practices. Examples of QC and QA, as described in various Medical Physics Technical Standards and Practice Parameters were discussed to provide the reader with a sense of where QC and QA fits in the overall structure of QM and QMS. While specific applications were presented for ACR Practice Parameters and Technical Standards, the concepts of QM, QA, and QC classification are generalizable to other guidance initiatives in medical radiological environments.

## AUTHOR CONTRIBUTIONS

All authors made substantial contributions to the design of the work, drafted and revised the content critically, approved the final version, and agree to be accountable for all aspects of the work, meeting ICMJE requirements for authorship.

The author(s) declare(s) that they had full access to all of the data in this study and the author(s) take(s) complete responsibility for the integrity of the data and the accuracy of the data analysis.

## CONFLICT OF INTEREST

M. Mahesh is Associate Editor and Medical Physics Editor of JACR. M. Mahesh and M. Amurao receive honoraria from ACR as accreditation program reviewers. All other authors declare no relevant conflicts of interest.

This project was unfunded; it is a work product of an ACR committee, and the result of a White Paper application approved by the Executive Committee of the ACR to be a position of the ACR.
